# Commentary: Effects of Whole Body Electrostimulation Associated With Body Weight Training on Functional Capacity and Body Composition in Inactive Older People

**DOI:** 10.3389/fphys.2021.719075

**Published:** 2021-07-16

**Authors:** Moacir Marocolo, Bernardo N. Ide, Mario Antonio Moura Simim, Luis Filipe Moutinho Leitão, Dustin J. Oranchuk, Clarkson P. C. Santos, Bruno V. C. Silva, Gustavo R. Mota

**Affiliations:** ^1^Physiology and Human Performance Research Group, Department of Physiology, Federal University of Juiz de Fora, Juiz de Fora, Brazil; ^2^Exercise Science, Health and Human Performance Research Group, Department of Sport Sciences, Institute of Health Sciences, Federal University of Triângulo Mineiro, Uberaba, Brazil; ^3^Physical Education and Adapted Sports Research Group, Institute of Physical Education and Sports, Federal University of Ceará, Fortaleza, Brazil; ^4^Superior School of Education, Polytechnic Institute of Setubal, Setubal, Portugal; ^5^Life Quality Research Centre, Rio Maior, Portugal; ^6^Sports Performance Research Institute New Zealand, School of Sport and Recreation, Auckland University of Technology, Auckland, New Zealand; ^7^Human Health and Performance Study and Research Group, Bahiana School of Medicine and Public Health, Salvador, Brazil; ^8^University Center of Belo Horizonte (Uni-BH), Belo Horizonte, Brazil

**Keywords:** exercise, electrostimulation, resistance training (strength), data analysis - methods, hypertrophy

## Introduction

Investigations about the influence of different training methods on muscle hypertrophy and neuromuscular function in the elderly are quite relevant to general population health (Garcia-Hermoso et al., 2018). Thus, we read the recent manuscript by Evangelista et al. (2021) about the effects of whole-body electrostimulation (WB-EMS) with body weight (BW) training on the functional fitness and body composition of older men. The final version of their manuscript was available on 1st April 2021 (See attached file). Afterwards, on 20th May 2021, a corrigendum was published (Evangelista et al., 2021). Even after the corrigendum, we found several inconsistencies. Additionally, since the study was supported by the manufacturer of the electrostimulation equipment (Xbody Brazil) (Evangelista et al., 2021) we think our critical comments deserve clarification. We believe this commentary will enrich the debate of such a relevant topic (i.e., exercise for the elderly population).

## Inconsistent Data Pattern

### Lean Body Mass

**Figure 1:**
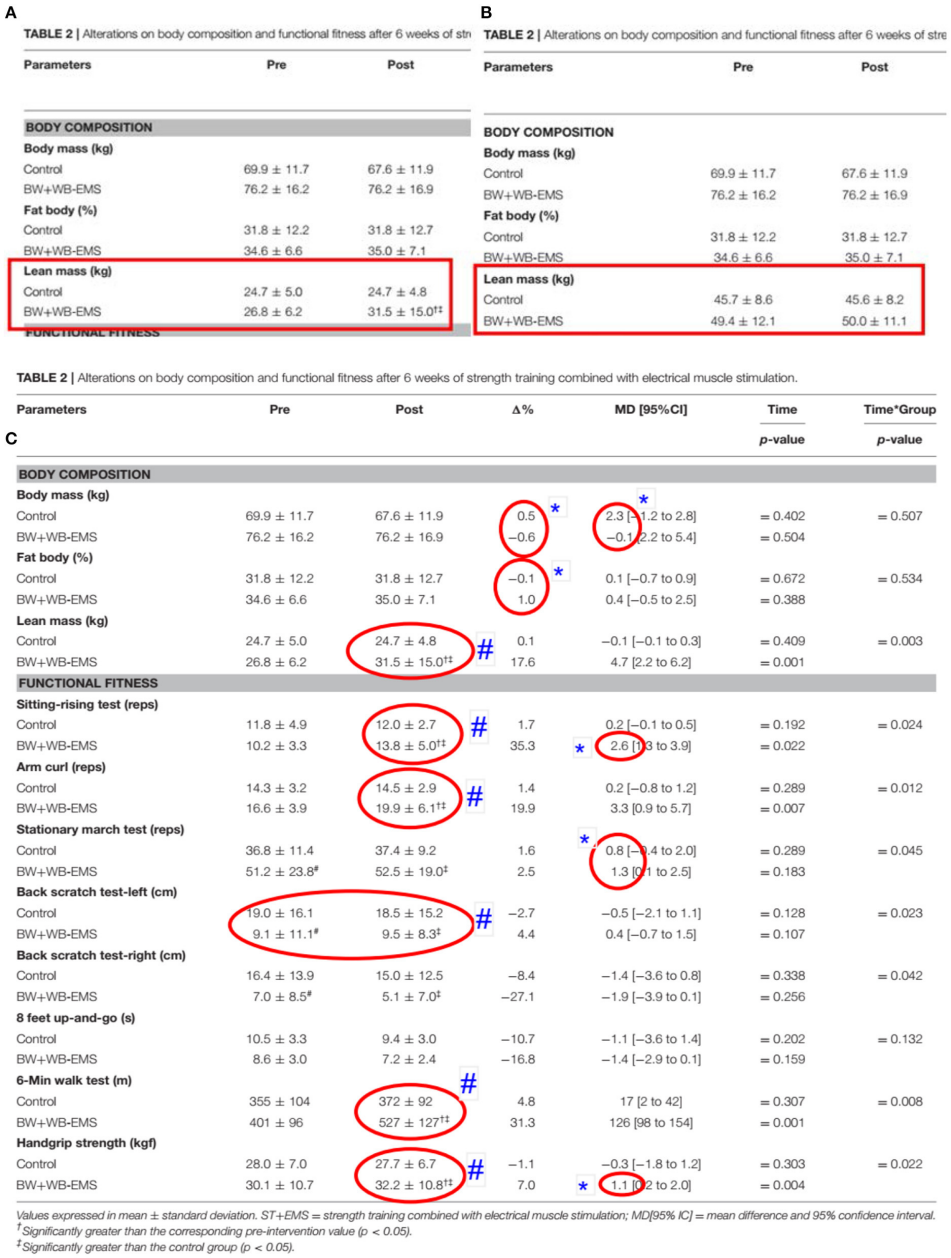
Indeed, the authors did not change “just the statistical signs” as they claimed, but all lean mass values as seen here: **(A)** Lean mass data presented on Table 2 of the first version of the article (Evangelista et al., 2021) available on 1st April; **(B)** Lean mass data presented on Table 2 post the corrigendum published on 20th May 2021 (Evangelista et al., 2021). **(C)** Table 2 of the first version of the article (Evangelista et al., 2021) available on 1st April 2021. Red circles indicate inconsistent data. ^*^Values not befitting; #results reported as significantly different, but our analysis showed that they are not.

Table 2, presented on the first version of the article, showed inconsistent body composition data for both groups at Pre and Post moments (See Figure 1). The control group reported pre-values for body mass, body fat, and lean mass of 69.9 ± 11.7 kg, 31.8 ± 12.2%, and 24.7 ± 4.8 kg, respectively.

According to their body fat data (31.8 ± 12.2%), the fat mass should be 22.2 ± 1.4 kg. Subtracting the calculated fat mass from the body mass reported resulted in a lean mass of 47.7 ± 10.3 kg (i.e., 69.9–22.2 kg). Although, the lean mass values reported by the authors were 24.7 ± 5.0 kg. There is a difference of values of 23 ± 5.3 kg! The same difference for body mass also occurred for the other BW+WB-EMS group (see Supplementary Material).

In the corrigendum version, there is still a difference in the values in Table 2 regarding lean mass signs †# indicating a statistically significant difference between Pre vs. Post moments. Not just the signs, but all lean mass values were modified! (See Figure 1). Unfortunately, the authors did not clarify that they also changed all absolute values, which may confound the readers about the corrections performed in the new version (i.e., in the corrigendum). Although, even in the corrigendum version, it is still lacking 2.0 and 0.4 kg in the Control and BW+WB-EMS group, respectively (see Supplementary Material).

### Muscle Thickness

The changes in vastus lateralis muscle thickness presented in Figures 4C,D of their article (Evangelista et al., 2021) have different range intervals for the same vastus lateralis muscle thickness. Figure 4C, presents individual increases of more than 100 mm (10 cm) in vastus lateralis muscle thickness, with one subject experiencing growth of nearly 400 mm (40 cm) while the same parameter in Figure 4D presents increase of no more than 10 mm! We do not believe this is an error of significant digits once an increase of even 4 cm is well-beyond the total mid-region vastus lateralis muscle thickness of ~2.8 cm in chronically resistance-trained young men (Oranchuk et al., 2019). These disproportionate numbers are highlighted further by the previously reported low typical errors of 1.6–6% for vastus lateralis muscle thickness (Oranchuk et al., 2019, 2021). Also, since neural adaptations are the most evident adaptation during the early stages of training in untrained subjects (Moritani and Devries, 1979; Sale, 1988; Jessee et al., 2021), all their results of muscle thickness are entirely outlier (i.e., only 6 weeks of training). Finally, the Y-axis of FigA, B, D, and E range from −10 to 15 mm, whereas panel C ranges from −100 to 500 mm, leading us to believe that there was an error in data transcribing, log-transformation, or similar.

### Statistical Analysis

Using mean, standard deviation, and sample size data provided in Table 2 of both versions of their article, we found a disagreement between reported results in their manuscript. Most of their data results are incorrect (please, see Figure 1C and the attached Excel and GraphPad Prism files).

In addition, the log transformation used by the authors may not be an appropriate statistical procedure (Feng et al., 2014). However, since their presented data are not in log scale but the original raw scale, those data do not support results and the study's conclusion. These differences may occur due to transformed mean values used by the authors, which evolve different parameters from the possible original data. Thus, we would like to ask if there was any problem with the data of the study. Furthermore, why was log transformation applied to data that generally presented normal distribution?

## Relevant Information Missing, and a Request for Raw Data

There is also relevant information missing: (1) participant's age; (2) warm-up exercises, intensity, and volume; (3) rest intervals between sets and sessions. As we wished to check their article for additional inconsistencies, as their article presents a data availability statement, we requested raw data from the corresponding author by email on 3rd April 2021 and by phone contact with other co-authors. However, until the submission of the current manuscript (01st June 2021), our request has not been addressed.

Additionally, in scientific articles, it is mandatory to show clearly methods used to allow anyone to reproduce the experiment. Unfortunately, this is not the case in the paper of Evangelista et al. (2021). Therefore, the following information should also be clarified in their original publication:

- the exercises performed are only named and not described. In order to reproduce the results, more precise information about the execution of the exercises should be given.
- the photos in Figure 2 seem to be of commercial nature and not taken by the authors. Therefore, the source of the pictures would have to be indicated. Lastly, the positioning of the electrodes on the body was not described and cannot be seen from Figure 2. This should also be added.

Also, we are concerned about the possible consequences of misinterpretations of the data by coaches, students, and the elderly population. According to the company's Brazilian website, the equipment used in their study has a cost of ~$20,000 USD, with a training session costing ~$30 USD, both representing a high cost for most Brazilians (i.e., higher than ~$150 BRL). The practice of traditional strength training, a simpler, more affordable, and accessible training method, is already established for inducing muscle hypertrophy and improving neuromuscular function in the elderly, likely preventing adverse events in older populations, including falls, fractures, and hospitalizations (Lavin et al., 2019). In addition to correcting the results, the high costs of the WB-EMS equipment should be highlighted as a limitation of this training method.

## Author Contributions

MM conceived the idea, wrote the first draft, worked on all drafts, and formatted the manuscript for submission. BI, MS, LL, DO, CS, BS, and GM helped on data analysis, and draft the paper. All authors read and approved the final version of the manuscript.

## Conflict of Interest

The authors declare that the research was conducted in the absence of any commercial or financial relationships that could be construed as a potential conflict of interest.
